# Maternal Cardiovascular Responses to Position Change in Pregnancy

**DOI:** 10.3390/biology12091268

**Published:** 2023-09-21

**Authors:** Alys R. Clark, Hanna Fontinha, John Thompson, Sophie Couper, Devanshi Jani, Ali Mirjalili, Laura Bennet, Peter Stone

**Affiliations:** 1 Auckland Bioengineering Institute, University of Auckland, Auckland 1010, New Zealand; 2 Department of Obstetrics and Gynaecology, Faculty of Medical and Health Sciences, University of Auckland, Auckland 1023, New Zealand; 3 Department of Anatomy and Medical Imaging, Faculty of Medical and Health Sciences, University of Auckland, Auckland 1023, New Zealand; 4 Department of Physiology, Faculty of Medical and Health Sciences, University of Auckland, Auckland 1023, New Zealand

**Keywords:** autonomic control, blood flow, cardiovascular adaptions, magnetic resonance imaging, pregnancy

## Abstract

**Simple Summary:**

Pregnancy requires major adaptions to blood circulation in the mother, because the developing baby needs an increasing supply of nutrients from the mother to grow. We know that this blood circulation can be influenced by position, for example, when the mother sits, stands or lies on her back. Some positions, like lying on the back, can increase risk of pregnancy loss. We present research that provides evidence for how blood supply is changed by both pregnancy and position. The primary aim of this work is to provide a description of what is expected in a normal pregnancy, so that we can provide a basis for future studies that investigate pregnancies that are not going as well as they should.

**Abstract:**

The maternal cardiovascular-circulatory system undergoes profound changes almost from the conception of a pregnancy until the postpartum period to support the maternal adaptions required for pregnancy and lactation. Maintenance of cardiovascular homeostasis requires changes in the cardiovascular autonomic responses. Here, we present a longitudinal study of the maternal cardiovascular autonomic responses to pregnancy and maternal position. Over a normal gestation, in the left lateral position there are significant changes in both time and frequency domain parameters reflecting heart rate variability. We show that cardiovascular autonomic responses to physiological stressors (standing and supine positions in late pregnancy) became significantly different with advancing gestation. In the third trimester, 60% of the subjects had an unstable heart rate response on standing, and these subjects had a significantly reduced sample entropy evident in their heart rate variability data. By 6 weeks, postpartum function returned to near the non-pregnant state, but there were consistent differences in high-frequency power when compared to nulligravid cases. Finally, we review complementary evidence, in particular from magnetic resonance imaging, that provides insights into the maternal and fetal impacts of positioning in pregnancy. This demonstrates a clear relationship between supine position and maternal hemodynamic parameters, which relates to compression of the inferior vena cava (*p* = 0.05). Together, these studies demonstrate new understanding of the physiology of physiological stressors related to position.

## 1. Introduction

A recent global assessment of stillbirth—the death of a baby before it is born—estimated around 2 million babies at 28 weeks or greater are stillborn, with a global rate of 13.9/1000 total births, with the highest rates seen in west and central Africa (22.8 per 1000 compared to 2.9/total births in Western Europe [1]. Stillbirth is one of the most traumatic events a family can experience [2], but it is recognized that improvements in maternal care, antenatal monitoring and application of growth charts tailored to specific populations can significantly reduce these rates [3]. However, to provide evidence-based guidelines and develop methods to detect those at risk, there is a need to better understand causal pathways [3,4]. It is striking that a significant proportion of cases, potentially around 20–60% of all cases, are of unknown origin [5].

Fetal growth restriction is difficult to accurately diagnose [6]. It is strongly associated with stillbirth and is considered the largest population-based attributable risk factor for preventing stillbirth [7]. However, many normally grown babies (growth is appropriate for gestational age) may also die before birth [8]. Hypoxia is considered the key terminal pathway in most cases of stillbirth [9]. It may occur secondarily to placental insufficiency and haemorrhage [10], and other factors may contribute, such as sleep-disordered breathing [11], maternal hyper- or hypotension [12], and sleep position with associated changes in uterine perfusion [7]. For example, maternal hypotension has been linked with an increased risk of stillbirth [12,13]. In late-gestation pregnancy, lying supine results in a decrease in maternal cardiac output due to compression of the inferior vena cava (IVC) [14] and is an independent but modifiable risk factor for stillbirth [7]. We have shown that fetal behaviour is affected by maternal position, with the fetus more likely to be in an energy-saving, quiescent state (behavioural state 1F) when the mother is supine compared with being in a left lateral position [15].

The maternal supine position is associated with supine hypotension in some women [16], termed, when symptomatic, the supine hypotension syndrome (SHS). Whether such women are at more risk of adverse pregnancy outcome, or conversely, that SHS is a protective response eliciting a manoeuvre to change position, is unclear. Changes in maternal circulatory function in pregnancy, and how maternal position influences these factors, can be assessed non-invasively by measurement of maternal heart rate and subsequent analysis of autonomic control [17,18]. In the current study, we describe a longitudinal study of maternal cardiovascular autonomic responses to position change during pregnancy that was undertaken to compare heart rate and blood pressure responses in nulligravid (never pregnant) young women aged 18 years and over, to a group of women followed throughout pregnancy and in the postpartum period. Alongside this longitudinal study, we review maternal autonomic changes in conjunction with magnetic resonance imaging (MRI) to facilitate the addition of functional information on the extent of maternal cardiovascular adaptions to pregnancy and the impact of maternal position on the function of the maternal cardiovascular system, was well as the functional implications for the placenta and fetus.

## 2. Materials and Methods

Here we describe a longitudinal study of maternal cardiovascular autonomic responses to position change during pregnancy undertaken to compare heart rate and blood pressure responses in nulligravid (never pregnant) young women aged 18 years and over, to a group of women followed throughout pregnancy and in the postpartum period.

The study was approved by the New Zealand Health and Disability Ethics Committee (HDEC 17/NTB/184). All subjects were aged 18 years and over, were non-smokers, normotensive, and had a non-pregnant body mass index (BMI) of <30, with no comorbidities. Pregnancies complicated by miscarriage, fetal abnormality, preeclampsia, or other antenatal obstetric complications were excluded. Gestation was confirmed by early pregnancy ultrasound, and the women were studied three times during pregnancy: at the end of the first trimester (12 weeks ± 2 weeks), in the second (25 weeks ± 2 weeks) and third (38 weeks ± 2 weeks) trimesters, and after the birth (6 ± 2 weeks post-partum).

**Figure 1:**
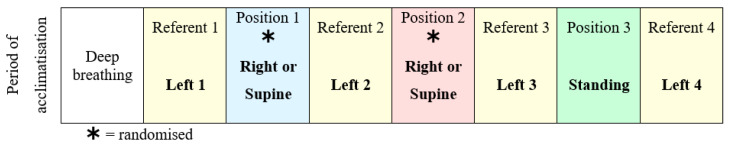
Data collection protocol used in each study visit, with right and supine positions randomized to reduce any order effect.

At each visit, the subjects underwent a protocol commencing with a minimum of 10 min of quiet rest for acclimatization, then deep breathing followed by position changes [19,20]. Deep breathing involved participants completing eight maximal inhalations and exhalations at a frequency of 0.1 Hz in time with a visual metronome. The last six full breaths were used for analysis. Flow and frequency were monitored using recordings via nasal cannula. The left lateral position was the referent position and was used as the comparator for right, supine, and standing. After the sequence illustrated in Figure 1, the subjects finally undertook an orthostatic manoeuvre, which is a movement from left lateral to standing, during which the baroreflex was assessed. The women remained standing for 8 min, during which heart rate and blood pressure were recorded. Deep breathing was performed between each position. Three minutes of recovery post-position change were allowed for stabilization before five minutes of recording were used for measurements in each position.

Maternal blood pressure was continuously measured at the fingers using the volume-clamp method via a CNAP^®^ monitor (CNSystems Medizintechnik AG) on the right index and middle fingers with a self-inflating sphygmomanometer cuff on the right upper arm for calibration. Continuous maternal heart rate was measured using an echocardiogram (ECG) with a sampling rate of 250 Hz, via three electrodes placed in a modified Einthoven triangle. Data were collected and analysed using customized software developed using LabVIEW^®^ (National Instruments, Austin, TX, USA, www.ni.com accessed on 1 July 2023). Standardized post-measurement correction factors were applied in the lateral positions to account for hydrostatic effects while the blood pressure cuff was not at the level of the heart [21,22]. In addition, to ensure fetal safety and record uterine activity, a continuous fetal ECG (Monica AN24^TM^ Monica Healthware, Nottingham, UK) was measured in the second and third trimester pregnancies for ambulatory fetal heart rate recording and assessment of uterine contractions.

The data acquired for analysis were heart rate, systolic blood pressure and RR intervals (the time between successive R waves). In the time domain, standard measures of heart rate variability (HRV) generally correlate closely with each other [17]. Hence, we have used (1), the standard deviation of all normal RR intervals in the recordings (SDNN), which measures the overall variability in the RR intervals and is primarily sourced from the parasympathetically driven respiratory sinus arrhythmia (RSA) at rest [23], and (2), the square root of the mean of sum of squares of differences between successive RR intervals in the recording (RMSSD). This reflects the variability between individual RR intervals and primarily represents parasympathetic modulation of heart rate [23]. Both SDNN and RMSSD are highly correlated with vagal control.

Time domain measures cannot separate different types of variation that contribute to HRV; hence, we used a frequency domain approach, with fast Fourier transformation to break the different rhythmic components of HRV into frequency bands [24]. Low-frequency (LF) and high-frequency (HF) power are normalized to total power and so reported in normalized units (nu), permitting these measures to be used to compare different subjects. The non-linear analyses are used to describe the irregularity of RR intervals within a given time frame [25]. The two approaches we used to explore the utility of non-linear measurement were sample entropy, a measure of the complexity or disorder in a system [26], and DFA, a type of fractal analysis which may quantify non-stationary or irregular behaviour of HRV. DFA α1 calculates short-term and α2 long-term exponents of HRV changes [25].

### Statistical Analysis

Comparison of maternal age and maternal anthropometry between the control and pregnant groups were undertaken by *t*-tests. To assess the effect of pregnancy (nulligravid versus early pregnancy data in the left lateral referent position), a univariable analysis using a *t*-test to compare means was used. Similarly, to compare outcomes of the left lateral position in mid- and late pregnancy to the left lateral position in early pregnancy multivariable analyses were used, these two tests are independent of each other.

To assess the effect of timing of pregnancy and maternal position, a multivariable repeated analysis was performed. The model included gestational trimester (categorized as early, mid-, and late) and position (categorized as left, right, and supine) with the participant ID used to identify the repeated measures. For each analysis an additional model was also used to assess an interaction between trimester and position).

Some women showed an unstable heart rate when standing. To analyse this effect, the cohort was split into two groups (those with stable and those with unstable characteristics). An independent sample *t*-test between groups was used to assess the significance of difference between groups, under the null hypothesis that the groups have equal means for each parameter. Levene’s test for equality of variances was used to assess variances between groups, and where variances did not meet the equality assumptions the Satterthwaite *t*-test for unequal variances was used.

For all analyses, statistical significance was determined as *p* < 0.05. Data are presented as mean and standard deviation or mean difference and 95% confidence interval.

## 3. Results

The study included 25 nulligravid (control women); 63 pregnant women completed the first trimester visit, 55 women undertook the second trimester visit, and 52 completed the investigations in the third trimester, with 49 women completing all three visits. Thirty-two women completed the postpartum visit, with 29 women having completed all four visits. All pregnancies delivered normally were live births without neonatal intensive care admission. Participant characteristics are summarized in Table 1.

Mean heart rate (HR) and blood pressure (BP) were significantly lower in each of the left lateral referent positions compared with the positions that followed them, and this effect was consistent throughout gestation with no interaction between the order in which positions changed and gestation. The mean difference in HR was 5 bpm (95% confidence interval (CI) 4–6) and in systolic BP −3 mmHg (95% CI −5 −1). Changes in some cardiovascular autonomic measurements, such as systolic blood pressure, SDNN and LF, were apparent from the first trimester while women were lying in the left lateral position, and there are further changes with advancing gestation while lying in the left lateral position (Table 2).

**Figure 2:**
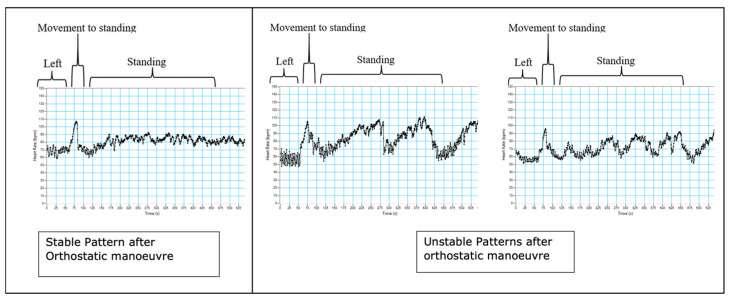
Examples of stable patterns (**left**) and unstable patterns (**middle** and **right**) after an orthostatic manoeuvre. These unstable patterns arose in the third trimester, with 60% of participants exhibiting unstable patterns at this stage in pregnancy.

During the orthostatic manoeuvre in the third trimester, 60% (26 of 42 participants) of the subjects had an unstable heart rate response on standing for the full duration of the recording over 8 min (examples are shown in Figure 2). This pattern is defined as heart rate fluctuations > 20 beats per minute whilst standing. This was not seen in the nulligravid group. A summary of all the cardiovascular autonomic responses found that the women with the unstable pattern had lower systolic blood pressure in left lateral and supine positions and when standing compared with the stable group (Table 3). Additionally, sample entropy was significantly different when the unstable group was supine and on standing. All other measures were not significantly different between the groups. There was no relationship between reported symptoms of supine hypotension in the two weeks prior to testing and heart rate pattern after the orthostatic manoeuvre.

Table 4 highlights significant changes in cardiovascular autonomic response with both position (standing, supine and right lateral with left lateral as the referent position) and gestation, highlighting independent changes and interactions between position and gestation. Numerical data showing absolute changes and *p*-values are provided as Supplementary Material. Regardless of position, women in mid- and late pregnancy demonstrated significantly different measures of autonomic function compared to women in early pregnancy. While there were some differences in autonomic response in right compared to left lateral positions, supine and upright positions showed a number of interactions between position and gestation, suggesting that these positions are stressors compared with lateral positions in pregnancy.

To assess the effect of pregnancy on each parameter of interest, we compared the nulligravid group to the post-partum group. In the left lateral position, there were no significant differences in mean heart rate (*p* = 0.70), mean systolic blood pressure, any time domain measures (RR mean, *p* = 0.58; SDNN, *p* = 0.21; RMSSD, *p* = 0.18), any non-linear measures (sample entropy, *p* = 0.62; α1, *p* = 0.08; α2, *p* = 0.71), or spontaneous baroreflex sensitivity (*p* = 0.28) between groups. Absolute high-frequency power (HF, ln), a frequency domain measure, was the only measure to demonstrate a statistically significant difference between the two groups, decreasing in postpartum women compared to nulligravid women (MD = −0.5, 95% CI = (−0.9, −0.1), *p* = 0.01). This change in HF was consistent in all data across all positions (left + right, *p* = 0.02, left + supine, *p* = 0.02), except standing (left + upright, *p* = 0.12).

## 4. Discussion

Our longitudinal study suggests that by the end of the first trimester, maternal cardiovascular autonomic measures are significantly different from those of a non-pregnant cohort. This indicates increased sympathetic and decreased parasympathetic contributions to cardiac rate control in early pregnancy. In a healthy pregnancy, maternal cardiac output increases progressively until mid-gestation, with smaller changes later in gestation [27]. The changes in heart rate observed in our data, beginning during the first trimester, saw cardiac output maintained as systemic vascular resistance fell [28], with a concurrent increase in vascular compliance [29]. The evidence obtained from the non-linear methods of HRV analysis support the increasing sympathetic activation beyond early pregnancy with modulation of cardiovagal activity without withdrawal of parasympathetic activity.

As gestation advanced, the response to supine positioning differed. In early to mid-pregnancy, supine and left lateral positions produced similar responses. However, in late pregnancy, the supine position became a physiologically stressful position reflected by differing cardiovascular autonomic responses compared to the left lateral position. Right and left lateral positions produced similar responses throughout pregnancy. The effects of maternal position on cardiac output are discussed in Section 3. Maternal cardiovascular autonomic responses whilst standing were already maximal in the first trimester and remained similar throughout the rest of the pregnancy. Combined with decreased HRV in left lateral position with advancing gestation, the magnitude of the autonomic response to standing from left lateral also decreased, suggesting that the ability of the autonomic nervous system to respond to the orthostatic stress as measured by heart rate and blood pressure is reduced as pregnancy advances [30].

A large subgroup of pregnant women In this study exhibited a markedly unstable heart rate whilst standing in the third trimester. The observation of an oscillating periodic tachycardia has also been reported by Schneider [31,32], who attributed this to intermittent interruption to femoral venous blood flow, possibly due to painless uterine contractions. Our studies showed that the periodicity was not consistent with the arterial baroreflex or respiratory sinus arrhythmia and no differences in uterine activity were seen between those with stable and unstable heart rates on standing. The sample entropy results are consistent with the concept of reduced vasoconstrictor reserve [33], and an upper limit to sympathetic activity in those with an unstable heart rate after the orthostatic manoeuvre. Further studies are needed to assess whether these women have an autonomic dysfunction which affects pregnancy outcome or whether the converse may apply and those with a stable heart rate, higher blood pressure when recumbent or in left lateral positions and a higher sample entropy result are more at risk of adverse pregnancy outcomes such as preeclampsia, fetal growth restriction or intrauterine death.

Overall, post-partum women demonstrate similar measures of cardiovascular autonomic function compared to nulligravid women. However, there is a difference in absolute high-frequency power, suggesting short-term (beat-to-beat) differences in function after 6 weeks post-partum and likely reflecting attenuated cardiovagal activity [34,35]. Prior literature has found that short-term maternal cardiovascular autonomic activity measured by changes in heart rate and blood pressure responses and sympathetic activity has returned to the non-pregnant state by 6 weeks post-partum [36,37,38], consistent with our findings. However, the finding that there were differences in autonomic function in post-partum versus nulligravid women suggests that future studies should carefully distinguish characteristics of pregnant and non-pregnant groups.

## 5. Review of Physiological Assessments of Maternal and Fetal Impacts of Pregnancy and Position

Evidence from our longitudinal study that maternal supine positioning is physiologically stressful for the mother motivates further physiological study to understand how the supine position influences maternal cardiovascular physiology. Here, we review complementary evidence of the impact of supine positioning on maternal-fetal physiology, using techniques that allow deeper interrogation of the cardiovascular changes associated with pregnancy, with the most recent evidence obtained from MRI.

### 5.1. Maternal

While the maternal cardiac output increases in the second half of pregnancy, the fetus grows, at a rate of >160 g/week [39]. The combined weight of the fetus and the gravid uterus are sufficient to apply a compression to the major maternal veins, which depends on maternal position. It is well established that compression of the IVC occurs in the supine position in late-gestation pregnancy [40,41]. The consequence of this compression is to reduce venous return within the maternal circulation. The mother must then compensate, with an aim to increase cardiac output. This is evident in our longitudinal data, reflecting that the supine position is a functional stressor in late pregnancy.

The functional consequence of IVC compression has been assessed by phase-contrast MRI, showing that in late-gestation pregnancy there is indeed a major decrease in blood flow in the IVC in supine compared to left lateral position [14,42]. There is evidence that there is between-participant variability, likely due to anatomical variations, between lVC volume with left or right lateral tilt from the supine position, with left tilt consistently leading to an increase in IVC volume [43]. In normal pregnancies, lying supine results in a 16% reduction in cardiac output and a 32% reduction in aortic blood flow at the bifurcation at the level where the internal iliac arteries arise to supply the uterine circulation [14]. Compensation for reduced IVC blood flow return to the heart is therefore only partially achieved through the collateral venous circulation and the azygos venous system [14]. These changes are evident in the heart, with MRI showing that there is an increased left ventricular end diastolic volume, ejection fraction, and stroke volume in supine compared to left lateral positions, with more moderate changes in the right heart [44]. It appears that the hemodynamic effect (measurable by MRI) of supine positioning is evident at mid-gestation with an increasing effect as the pregnancy continues toward term [42,44].

A study of pregnancies with symptomatic supine hypotension [45] suggested that there was a significant reduction in blood flow through the azygos vein in these pregnancies with no other differences in blood flow compared to a control group. This suggested that there may be anatomical differences in the azygos vein that might contribute to the symptoms of supine hypotension. The azygous system provides one of the main alternative routes for blood flow during IVC compression but is highly anatomically variable. However, studies investigating the anatomy of this system may provide an avenue to investigate potential risk of this syndrome, acknowledging that our autonomic studies suggest that there may be a group of pregnancies that are asymptomatic but at risk of the effects of supine hypotension.

One limitation to understanding the maternal cardiovascular response to pregnancy is that while measurement of heart rate responses to stressors provides a non-invasive indicator of autonomic function, it cannot identify the internal redistribution of blood supply due to the change in position. MRI is able to assess these effects, but it is limited to identification of flow, or flow patterns, in a small number of arteries or veins within the circulation. Computational hemodynamic assessments could potentially link these two modes of measurement and help to identify features that could contribute to observed differences between control groups and those with supine hypotension (with or without symptoms). Indeed, whole-circulation computational models have been successfully used to predict short- (<5 min) and longer-term (>5 min) responses to orthostatic stressors, but only in the non-pregnant context [46]. Similar whole-body models have been developed for pregnancy, but with a focus on identifying features of pathological vascular adaption in pre-eclamptic pregnancies [47,48]. Similar models, parameterized to personalized imaging features (e.g., from MRI), may help to determine whether anatomy of the venous circulation might contribute to observed differences between groups in cardiovascular adaption to position in the second half of pregnancy.

### 5.2. Utero-Placental Adaption

While maternal cardiac output increases by approximately 30% in pregnancy, the blood flow to the uterus via the uterine arteries increases to approximately 15 times its non-pregnant rate over the course of gestation, with most of this increase occurring following the first trimester [49]. In the first trimester, the placenta develops in a physiologically hypoxic environment, with maternal blood supply to the placental surface limited to plasma percolation through trophoblastic plugs that fill the lumen of the spiral arteries at the interface between the uterus and the placenta [50,51]. Some remnants of these plugs remain until mid-gestation, but progressively wider channels form, allowing a significantly increasing blood flow directly to the placental surface from 12–20 weeks [52]. All of the arteries in the uterus remodel in response to pregnancy, with an approximately doubling in size from 6 weeks of gestation to mid-gestation [52]. Importantly a network of arterio-venous anastomoses (shunt pathways) lie within the myometrium, hypothesized to be protective against maternal stressors (e.g., hypertension, or rapid increases in cardiac output) [53].

Late-gestation MRI studies conducted by our research group have assessed the impact of maternal position on the internal iliac arterial blood flow and delivery of blood and oxygen to the placenta [54]. Blood flow was quantified, using phase-contrast MRI images acquired from the right and left internal iliac arteries in supine and left lateral decubitus positions and the fetal umbilical vein in the same participants. Healthy pregnant women were scanned in both left lateral and supine positions between 34 and 38 weeks of gestation. Total internal iliac blood flow, which is the main blood supply to the uterus, is reduced by 23.7% when the woman moves from left lateral to supine position, consistent with the magnitude of changes in the aortic bifurcation in late pregnancy reported in previous studies [14]. This suggests that supine positioning is a stressor not only to the mother, but also to the fetus due to reduced delivery of nutrient rich blood to the placental surface. Uterine artery flow has been measured using phase-contrast MRI in pregnancy, but the uterine artery has significant anatomical variation [55,56], and so tracing this anatomy to determine the uterine artery anatomy and thus flow is not always feasible in studies that aim to take multiple measures of function in the same individual. However, in comprehensive imaging studies where different participants are grouped by imaged position [57], there is a consistent significant decrease in measured uterine artery flow from left lateral to supine that is comparable to that measured in internal iliac arteries [54].

How the reduction in blood flow to the uterus and placenta in supine position translates exactly to changes in blood flow to the placental surface via the myometrium is not yet fully studied. Blood delivery to the placental surface is influenced in volume and in speed by the effective remodelling of the uterine vasculature. The blood flow in the uterine artery itself is also not directly related to placental surface flow, in part due to the functional influence of arterio-venous anastomoses in the myometrium [58]. Venous compression at the IVC will influence venous pressure within the uterus itself, which may influence the delivery of blood through the utero-placental circulation, and is understudied at present.

### 5.3. Feto-Placental and Fetal Circulation

In addition to the maternal cardiovascular effects of position, our group studied the impact upon the fetus of the reduction in lower aortic blood flow and cardiac output when the mother turned supine. The study involved recruiting 160 pregnant women in the third trimester and recording beat-to-beat fetal heart rate continuously overnight [15]. Maternal position as determined by polysonography using Embletta gold^®^ devices had been validated by infrared video recordings in a cohort of 30 women. Two observers independently scored the fetal heart rate recordings to determine fetal behavioural states, which were then correlated with the maternal position [15]. It was found that when the mother was supine, fetal active state 4F was infrequent; conversely, quiescent state 1F was significantly more likely. Compared with the most common state (2F) in the supine position, the fetus was more likely to be in 1F (OR = 4.99, 95% CI 2.41, 10.43). These findings are consistent with the concept that the fetus can adapt to a hypoxic stressor by assuming a lower oxygen-consuming state [59].

Whilst the healthy placenta and fetus may tolerate the effects of up to a 50% reduction in uterine arterial blood flow [60], this is less likely to be the case in pregnancies complicated by maternal hypertensive disease and/or fetal growth restriction. In an overnight sleep study of 116 participants synchronizing maternal position with fetal heart rate events, maternal position change and especially in the supine position was significantly more likely to be associated with adverse FHR events in women with FGR and/or a hypertensive disorder compared with uncomplicated pregnancies (*p* = 0.006) [61], providing further evidence that the fetus was responding to hypoxia induced by position change.

**Figure 3:**
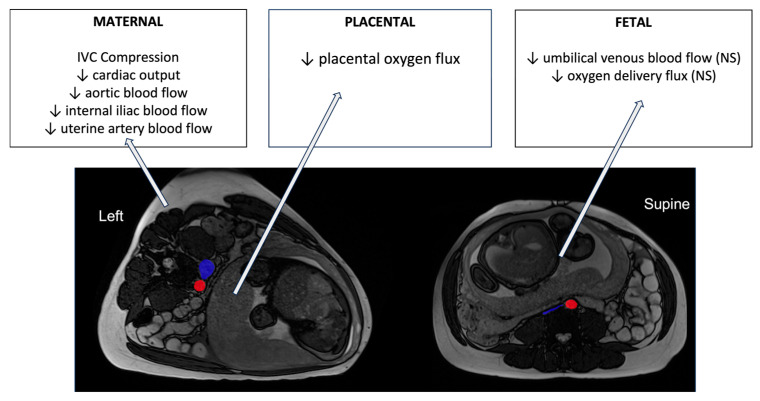
A summary of the changes in blood flow and oxygen transfer parameters in healthy pregnancies in late gestation derived from MRI imaging studies reviewed here. MRI images are shown in axial planes, using radiological convention for axes. The IVC and aorta are highlighted in blue and red, respectively. NS = non-significant changes.

To assess oxygen transfer across the placenta associated with the maternal blood flow changes in each of the maternal positions, a recently designed protocol termed DECIDE [62], which combined diffusion-weighted imaging (acquired at seven b-values) and T2 imaging (10 echo times), was used. The DECIDE protocol both separates maternal from fetal compartments of the placenta and quantifies the oxygen saturation in fetal blood in the areas of interest. To understand the impacts of the supine position on the placenta and the fetus, the DECIDE protocol has been combined with phase-contrast MRI in the same participants, imaged in both left lateral decubitus and supine positions [54]. Two physiological markers of placental function to indicate oxygen movement within the placenta and from placenta to fetus were derived from the phase-contrast and DECIDE parameters. These markers were termed placental and delivery (or oxygen delivery) flux. It was found that placental flux showed a statistically significant difference between supine and left lateral positions. The placental flux index reduced by 6.2% (*p* = 0.0380) when women were supine compared to LLD (0.000976 ± 0.00021 supine vs. 0.001064 ± 0.00020 LLD). The delivery flux index reduced by 11.2% when women lay supine compared to LLD (from 199.38 ± 60.70 LLD to 168.62 ± 48.22 supine), but this change did not reach significance (*p* = 0.0597) in these normal healthy pregnancies. Combined with phase-contrast imaging in the internal iliac arteries, this placental imaging suggests that the significant reduction in blood delivery to the uterus observed with supine positioning does reduce the flux of oxygen across the placenta, even in normal pregnancies, and even though this is well tolerated it does appear that a reduction in umbilical venous flow may occur, consistent with the trends observed in FHR data of increased likelihood of a fetus being in a quiescent state when the mother is supine (see Figure 3).

To understand further whether there is a direct link between IVC compression and reduction in placental oxygen delivery to the fetus, we revisited the imaging obtained by Couper et al. and quantified the cross-sectional area of the IVC and aorta at a position 12 mm superior to the aortic bifurcation in each participant imaged. We found a significant difference in aorta cross-sectional area from left lateral to supine positions (149.0 ± 30.7 mm^2^ in left lateral to 127.9 ± 25.0 mm^2^, *p* = 0.022), which is consistent with observed reductions in aortic and internal iliac flow rates between these two positions [14,54]. The compression of the IVC was significant in most participants (228.6 ± 133.3 mm^2^ in left lateral to 61.2 ± 19.2 mm^2^ in supine, *p* < 0.001), although the relatively larger variability in left lateral reflects that there were some cases where there was some IVC compression even in the left position. There was a relationship between the percentage decrease in the IVC from left lateral to supine, and the percentage decrease in total internal iliac flow from left lateral to supine (r = 0.19, *p* = 0.05), which reflects that in cases of more complete IVC compression, the ability for the mother to maintain cardiac output is likely restricted. However, there was significant variability in this relationship, and no significant relationship between the extent of IVC compression and any placental or fetal metric derived from MRI. This indicates that tolerance of IVC compression is likely pregnancy-specific, and some pregnancies may be more at risk than others.

Whilst the changes due to supine positioning may be tolerated in normal healthy pregnancy, as has been found in sheep studies, where the effect of reducing uterine blood flow by up to 30% (similar to the expected change with supine position indicated in MRI) appears not to cause circulatory redistribution in the fetus [63], this is less clear in pregnancies affected by fetal growth restriction. In an ongoing study comparing normal with growth-restricted pregnancy, using a similar MRI DECIDE Protocol as described, we have shown significant reductions in oxygen delivery to the growth restricted fetus when the mother changes from left lateral to supine positions. The changes in umbilical venous blood flow suggest circulatory redistribution in the fetus, either in the placental or fetal systemic circulations.

## 6. Conclusions

Adaptations in the maternal cardiovascular autonomic responses to normal day-to-day activities, including position change, are required to maintain sufficient blood pressure and utero-placental perfusion for the developing pregnancy. Standardized research protocols, which include a left lateral position and response to a positional challenge (be that moving to supine or standing positions), are particularly important in determining the normal changes expected in pregnancy and in future studies of pathological pregnancies. We have developed a suite of studies including heart rate variability and magnetic resonance imaging studies that allow quantification of the adaption of the maternal circulation to pregnancy and position. Data between studies of maternal heart rate variability, fetal behaviours, and imaging studies are consistent in their outcomes, in that they suggest that the maternal hemodynamic consequences of changing position (particularly to supine and upright) are significant, and fetal responses to supine positioning suggest a movement toward a more quiescent state, albeit a tolerated change in normal pregnancies.

Together these studies provide a description of normal function in pregnancy, but variability between pregnancies in terms of response suggests strongly that understanding what exactly defines normal is still a gap that exists in pregnancy research of this type. However, these data provide a strong basis for future studies of at-risk groups, including pregnancies with supine hypotensive disease or those impacted by fetal growth restriction.

## Figures and Tables

**Table 1:** Participant characteristics.

	Control Group (*n* = 25)		Pregnant Group (*n* = 63)		
	Mean	SD [Range]	Mean	SD [Range]	*p*-Value
Age (years)	28	4 [21–37]	32	4 [24–41]	<0.001
Height (cm)	166	7 [148–180]	167	8 [148–186]	0.68
Non-pregnant BMI (kg/m^2^)	23	3 [17–30]	23	3 [18–30]	0.68
Gestation at birth (days)			277	7 [260–293]	n/a
Birthweight (g)			3497	493 [2185–4450]	n/a

**Table 2:** Effect of pregnancy (early pregnancy compared to nulligravid) on cardiovascular autonomic responses in the first left lateral decubitus referent position.

First Left Lateral Position	Nulligravid		Early Pregnancy		Mid-Pregnancy		Late-Pregnancy		Effect of Pregnancy		Effect of Gestation		
	(N)		(T1)		(T2)		T3		Mean Difference (95% CI)		Mean Difference (95% CI)		
	Mean	SD	Mean	SD	Mean	SD	Mean	SD	T1-N	*p*	T2-T1	T3-T1	*p*
Heart rate and systolic blood pressure													
Mean heart rate (bpm)	62	10	66	7	72	7	73	10	3 (−1, 7)	0.18	**6 (4, 7)**	**8 (6, 9)**	**<0.001**
SBP (mmHg)	107	15	99	10	97	11	99	11	−**8 (**−**15,** −**2)**	**0.01**	−2 (−6, 1)	0 (−4, 3)	0.37
Time domain measures of HRV													
RR mean (ms)	993	170	921	104	842	80	837	127	−63 (−129, 3)	0.06	−**79 (**−**99,** −**59)**	−**88 (**−**111,** −**65)**	**<0.001**
SDNN (ms)	75	38	55	26	46	18	46	25	−**18 (**−**34, 3)**	**0.02**	−**9 (**−**15,** −**4)**	−**11 (**−**16,** −**5)**	**0.001**
RMSSD (ms)	70	49	52	35	38	24	38	33	−15 (−36, 6)	0.16	−**13 (**−**22,** −**5)**	−**15 (**−**22,** −**8)**	**<0.001**
Frequency domain measures of HRV													
HF (ln, ms^2^)	3.1	0.6	2.9	0.6	2.6	0.6	2.5	0.6	−0.2 (−0.5, 0.1)	0.19	−**0.3 (**−**0.4,** −**0.1)**	−**0.4 (**−**0.6,** −**0.3)**	**<0.001**
HF (nu)	44	21	48	20	42	22	41	22	5 (−6, 15)	0.36	−4 (−9, 0)	−7 (−12, −2)	0.02
LF (ln, ms^2^)	3	0.5	2.7	0.5	2.6	0.5	2.4	0.6	−**0.3 (**−**0.6, 0.0)**	**0.03**	−**0.2 (**−**0.3,** −**0.1)**	−**0.4 (**−**0.5,** −**0.2)**	**<0.001**
LF (nu)	35	17	33	16	35	16	31	16	−3 (−11, 6)	0.55	0 (−4, 4)	−3 (−7, 0)	0.1
Non-linear measures of HRV													
Sample entropy	1.55	0.3	1.55	0.28	1.41	0.38	1.47	0.37	0 (−0.15, 0.15)	0.98	−**0.13 (**−**0.23,** −**0.02)**	−**0.07 (**−**0.16, 0.03)**	**0.05**
α1	0.89	0.3	0.89	0.26	0.98	0.28	1	0.33	0 (−0.14, 0.14)	0.99	**0.08 (0.01, 0.14)**	**0.1 (0.03, 0.17)**	**0.02**
α2	0.86	0.23	0.79	0.2	0.85	0.24	0.91	0.22	−0.08 (−0.19, 0.03)	0.14	**0.05 (**−**0.01, 0.11)**	**0.13 (0.07, 0.19)**	**<0.001**
Spontaneous baroreflex sensitivity													
Spontaneous BRS	0.41	0.29	0.32	0.24	0.27	0.22	0.36	0.38	−0.08 (−0.23, 0.07)	0.32	−0.05 (−0.14, 0.04)	0.06 (−0.08, 0.20)	0.16

bpm = beats per minute; SBP = systolic blood pressure; mmHg = millimetres of mercury; HRV = heart rate variability; ms = milliseconds; nu = normalised units; RR = RR interval between successive R-waves (RR interval); SDNN = standard deviation of RR intervals; RMSSD = square root of the mean of the sum of all the squares of differences between adjacent normal RR intervals; HF = high frequency; ln = natural logarithm; LF = low frequency; BRS = baroreflex sensitivity; IIHR = initial increase in heart rate; max/min = maximum/minimum. Values which reached statistical significance are shown in bold.

**Table 3:** Cardiovascular autonomic responses to standing in women in the third trimester with and without stable standing heart rate patterns.

Standing	Stable		Unstable		Mean Difference (95% CI)	
	(S)		(U)			
	Mean	SD	Mean	SD	U-S	*p*
Heart rate and systolic blood pressure						
Mean heart rate (bpm)	86	9	90	9	4 (−2, 9)	0.18
SBP (mmHg)	117	16	112	11	−5 (−12, 3)	0.23
Time domain measures of HRV						
RR mean (ms)	707	83	681	75	−26 (−70, 19)	0.25
SDNN (ms)	51	24	64	30	13 (−2, 29)	0.1
RMSSD (ms)	32	39	27	21	−5 (−22, 12)	0.56
Frequency domain measures of HRV						
HF (ln, ms^2^)	2.3	0.6	2.4	0.6	0.1 (−0.2, 0.4)	0.57
HF (nu)	20	15	17	14	−3 (−12, 5)	0.44
LF (ln, ms^2^)	2.7	0.5	2.8	0.4	0.1 (−0.1, 0.4)	0.4
LF (nu)	39	18	33	15	−6 (−16, 3)	0.19
Non-linear measures of HRV						
Sample entropy	1.03	0.42	0.83	0.29	−**0.2 (**−**0.39, 0.00)**	**0.05**
α1	1.25	0.37	1.36	0.3	0.11 (−0.07, 0.30)	0.23
α2	1.05	0.27	1.15	0.22	0.1 (−0.03, 0.24)	0.14
Spontaneous baroreflex sensitivity						
Spontaneous BRS	0.34	0.17	0.47	0.33	0.13 (−0.06, 0.31)	0.12
Position change reflexes						
IIHR	33	9	38	9	**5 (0, 11)**	**0.05**
max/min	1.4	0.3	1.5	0.2	0.1 (−0.1, 0.2)	0.37

bpm = beats per minute; SBP = systolic blood pressure; mmHg = millimetres of mercury; HRV = heart rate variability; ms = milliseconds; nu = normalised units; RR = interval between successive R-waves (RR interval); SDNN = standard deviation of RR intervals; RMSSD = square root of the mean of the sum of all the squares of differences between adjacent normal RR intervals; HF = high frequency; ln = natural logarithmic; LF = low frequency; BRS = baroreflex sensitivity; IIHR = initial increase in heart rate; max/min = maximum/minimum. Values which reached statistical significance are shown in bold.

**Table 4:** Summary of statistically significant effects of gestation and effects of position on cardiovascular autonomic variables in women in early, mid-, and late pregnancy in left (L), right (R), supine (S), and standing (St) positions. Significant increases in a parameter are shown by green upward arrows, and significant decreases in a parameter are shown as red downward arrows. Where there are significant interactions between a parameter and gestation/position, these are shown as orange stars. A complete database of absolute changes and *p*-values are provided as Supplementary Material.

	Effect of Gestation (St and L Data Combined)		Effect of Position	Effect of Gestation (S and L Data Combined)		Effect of Position	Effect of Gestation (R and L Data Combined)		Effect of Position
	T2-T1	T3-T1	St-L	T2-T1	T3-T1	S-L	T2-T1	T3-T1	R-L
Heart rate and systolic blood pressure									
Mean heart rate (bpm)	*	*	*	↑	↑	-	↑	↑	↑
SBP (mmHg)	-	↑	↑	-	↑	↑	*	*	*
Time domain measures of HRV									
RR mean (ms)	*	*	*	**↓**	**↓**	-	**↓**	**↓**	**↓**
SDNN (ms)	*	*	*	**↓**	**↓**	-	**↓**	**↓**	-
RMSSD (ms)	*	*	*	*	*	*	**↓**	**↓**	-
Frequency domain measures of HRV									
HF (ln, ms^2^)	*	*	*	**↓**	**↓**	-	**↓**	**↓**	-
HF (nu)	*	*	*	*	*	*	**↓**	**↓**	-
LF (ln, ms^2^)	-	-	-	**↓**	**↓**	-	-	-	-
LF (nu)	*	*	*	-	-	-	*	*	*
Non-linear measures of HRV									
Sample entropy	-	**↓**	**↓**	*	*	*	**↓**	-	-
α1	*	*	*	*	*	*	↑	↑	-
α2	*	*	*	↑	↑	↑	-	↑	-
Spontaneous baroreflex sensitivity									
Spontaneous BRS	*	*	*	-	-	**↓**	-	-	**↓**
Position change reflexes									
IIHR	**↓**	**↓**		**↓**	**↓**		-	-	
max/min	↑	-		-	-		**↓**	**↓**	

bpm = beats per minute; SBP = systolic blood pressure; mmHg = millimetres of mercury; HRV = heart rate variability; ms = milliseconds; nu = normalised units; RR = interval between successive R-waves (RR interval); SDNN = standard deviation of RR intervals; RMSSD = square root of the mean of the sum of all the squares of differences between adjacent normal RR intervals; HF = high frequency; ln = natural logarithm; LF = low frequency; BRS = baroreflex sensitivity; IIHR = initial increase in heart rate; max/min = maximum/minimum.

## Data Availability

The data presented in this study are available on request from the corresponding author. The data are not publicly available due to ethical constraints.

## Supplementary Materials

The following supporting information can be downloaded at: https://www.mdpi.com/article/10.3390/biology12091268/s1.
